# Ratiometric Measurements of Adiponectin by Mass Spectrometry in Bottlenose Dolphins (*Tursiops truncatus*) with Iron Overload Reveal an Association with Insulin Resistance and Glucagon

**DOI:** 10.3389/fendo.2013.00132

**Published:** 2013-09-20

**Authors:** Benjamin A. Neely, Kevin P. Carlin, John M. Arthur, Wayne E. McFee, Michael G. Janech

**Affiliations:** ^1^Department of Medicine, Division of Nephrology, Medical University of South Carolina, Charleston, SC, USA; ^2^Translational Medicine and Research Program, National Marine Mammal Foundation, San Diego, CA, USA; ^3^Ralph H. Johnson Veterans Affairs Medical Center, Charleston, SC, USA; ^4^NOAA’s Ocean Service, National Centers for Coastal Ocean Science, Center for Coastal Environmental Health and Biomolecular Research, Charleston, SC, USA

**Keywords:** parallel reaction monitoring, marine mammal, assay, hemochromatosis, liver, diabetes

## Abstract

High molecular weight (HMW) adiponectin levels are reduced in humans with type 2 diabetes and insulin resistance. Similar to humans with insulin resistance, managed bottlenose dolphins (*Tursiops truncatus*) diagnosed with hemochromatosis (iron overload) have higher levels of 2 h post-prandial plasma insulin than healthy controls. A parallel reaction monitoring assay for dolphin serum adiponectin was developed based on tryptic peptides identified by mass spectrometry. Using identified post-translational modifications, a differential measurement was constructed. Total and unmodified adiponectin levels were measured in sera from dolphins with (*n* = 4) and without (*n* = 5) iron overload. This measurement yielded total adiponectin levels as well as site specific percent unmodified adiponectin that may inversely correlate with HMW adiponectin. Differences in insulin levels between iron overload cases and controls were observed 2 h post-prandial, but not during the fasting state. Thus, post-prandial as well as fasting serum adiponectin levels were measured to determine whether adiponectin and insulin would follow similar patterns. There was no difference in total adiponectin or percent unmodified adiponectin from case or control fasting animals. There was no difference in post-prandial total adiponectin levels between case and control dolphins (mean ± SD) at 763 ± 298 and 727 ± 291 pmol/ml, respectively (*p* = 0.91); however, percent unmodified adiponectin was significantly higher in post-prandial cases compared to controls (30.0 ± 6.3 versus 17.0 ± 6.6%, respectively; *p* = 0.016). Interestingly, both total and percent unmodified adiponectin were correlated with glucagon levels in controls (*r* = 0.999, *p*  < 0.001), but not in cases, which is possibly a reflection of insulin resistance. Although total adiponectin levels were not significantly different, the elevated percent unmodified adiponectin follows a trend similar to HMW adiponectin reported for humans with metabolic disorders.

## Introduction

Managed dolphins have higher serum ferritin levels, total serum iron levels, percent transferrin saturation, and total iron binding capacity compared to free-ranging dolphins (1). Managed dolphins are also susceptible to hemochromatosis (iron overload) where iron deposition in Kupffer cells is a prevalent diagnostic feature (2). While the cause of iron accumulation or iron overload is not known, elevated post-prandial insulin levels in dolphins were associated with excessive hepatic iron deposition (3). Humans with elevated iron stores but without hemochromatosis can have clinical laboratory values consistent with metabolic syndrome and insulin resistance (4), and hepatic iron overload is associated with insulin resistance regardless of the extent of liver damage (5).

Adiponectin is an adipokine primarily secreted by adipocytes and is considered an insulin-sensitizing hormone. Low circulating levels of adiponectin are associated with hyperinsulinemia (6), high liver lipid content, and obesity (7). Recently, iron was implicated in the direct negative regulation of adiponectin production by adipocytes which resulted in insulin resistance in mice (8) suggesting that the relationship between iron, adiponectin, and insulin sensitivity are tightly intertwined. Previous findings of elevated 2 h post-prandial serum insulin and glucagon levels in dolphins with hemochromatosis compared to controls, suggest that dolphins with iron overload may be unable to properly regulate insulin levels (3); however it is not known whether this observed post-prandial insulin and glucagon response is correlated with total serum adiponectin.

Reports of blood adiponectin levels in marine mammals are limited to a single species, Northern elephant seals (*Mirounga angustirostris*), and were measured in pups using a canine ELISA assay (9, 10). Interestingly, the mean levels reported were one to two orders of magnitude lower than mean levels reported for dogs (11–13), suggesting that marine mammals may have low circulating adiponectin levels, Northern elephant seals have low adiponectin levels, or that the ELISA assay utilized had limited cross-reactivity to elephant seal adiponectin. To assess true cross-reactivity of an antibody, a fully recombinant adiponectin protein is required, which is not commercially available for marine mammals. Cross-reactivity concerns can be avoided by utilizing an alternative mass spectrometry-based direct detection approach, parallel reaction monitoring [PRM; (14, 15)]. This method is similar to selected reaction monitoring (SRM) with the exception that all product ions from a collision induced dissociation reaction are monitored and available for extraction (16).

Adiponectin exists as multimers, commonly referred to as high and low molecular weight (HMW and LMW). Multimerization is largely governed by post-translational modifications (PTMs) within the collagenous domain of the protein and glycosylation following lysine hydroxylation is reported to be key in the formation of multimers as well as the ability to properly phosphorylate AMP-kinase in liver tissue (17). Because HMW adiponectin is best associated with diabetes and metabolic syndrome (18, 19) and because glycosylation of lysine is an important modification that drives intracellular AMP-kinase signaling, a new method is presented to provide a differential measurement of tryptic adiponectin peptides that could reflect some degree of lysine modification to serve as a proxy of adiponectin modification and possibly multimerization. This method relies on the differential measurement of total adiponectin and unmodified Lys75 adiponectin peptides to investigate whether degree of modification is associated with outcome data. Therefore, it was hypothesized that the fraction of total adiponectin that is unmodified at a known lysine (Lys75) would be elevated in iron overload dolphins at the 2 h post-prandial time point compared to controls. Additionally, it was hypothesized that 2 h post-prandial levels of total adiponectin would be lower compared to a control group of dolphins.

## Materials and Methods

### PRM method development serum

A single dolphin serum was used for PRM assay development, taken from a 2.44 m long female bottlenose dolphin (SC-0145). The dolphin was captured, sampled, and released on June 26, 2001 during a NOAA sponsored rescue and relocation operation of two dolphins located in a fresh water reservoir near Charleston, South Carolina. Both dolphins had moved into the freshwater reservoir on June 6, 2001. The sample was stored at −80°C. Before processing further, the serum was thawed at 37°C for 2–5 min, before being aliquoted into 110 μl aliquots which were frozen at −80°C. These aliquots were used fresh daily by thawing at 37°C for 1 min and mixed briefly by vortexing before use.

### Iron overload case-control study population

The study population consisted of nine bottlenose dolphins at the U.S. Navy Marine Mammal Program (MMP), including six females and three males with ages ranging from 8 to 48 years. The study included four dolphins with iron overload (cases) and five controls. Iron overload cases in this study were previously reported to have chronically elevated serum aminotransferases, specifically alanine aminotransferase (ALT) and aspartate aminotransferase (AST), as well as elevated serum iron (20). These four case animals had since undergone phlebotomy treatments in order to manage iron overload, and diagnoses of iron overload have been confirmed by live liver biopsies in three of the four animals (21). Control animals had normal blood values throughout the year before sample collection, specifically iron, ALT, and AST, based upon published reference ranges for healthy bottlenose dolphins (22). Of these control animals, one female surrogate was lactating and nursing a calf and another was in the early stage of pregnancy during the 2 h post-prandial phase.

### Iron overload case-control study population diet

Marine Mammal Program dolphins were fed high-quality, frozen-thawed fish and mollusks, including capelin (*Mallotus villosus*), herring (*Clupea harengus*), mackerel (*Scomber scombrus*), and squid (*Loligo brevis*). They received daily vitamin supplementation that did not include iron, as well as periodic anti-parasitic medications for prophylaxis. Proximate analysis was used to assess nutrient concentration in fish fed during the study period. Crude protein concentration was determined at Michelson Laboratories, Inc. (Commerce, CA, USA) using standard methods (23). Fed protein was calculated using the protein concentration reported by Michelson Labs along with daily food intake and animal weight. Measurements for age, weight, and fed protein were based on collection dates from 2 h post-prandial samples. If no length data was available on the weight date, the nearest length measurement was used. BMI was calculated using the standard human equation: weight per square length (kilogram per square meter).

### Iron overload case-control study sample collection

Marine Mammal Program dolphins are trained to voluntarily allow collection of blood from the ventral fluke veins. Blood was collected in BD Vacutainer (Rutherford, NJ, USA) blood collection tubes using 20- or 21-gage, 1.5″ needles. Blood was collected in ethylenediaminetetraacetic acid (EDTA), serum separator tubes (SST), and red top tubes (no additive). One vial each of serum and EDTA whole blood were submitted to Quest Diagnostic Laboratories (San Diego, CA, USA) or the Naval Medical Center (San Diego, CA, USA) for analysis of serum chemistry and complete blood count (CBC). Remaining blood was centrifuged at 3000 rpm for 10 min and separated prior to archiving. Serum and plasma samples were stored frozen at −80°C until analysis. EDTA plasma samples were processed and frozen within 30 min of blood collection. Plasma was analyzed for insulin and glucagon at Esoterix Inc., Laboratory Services (Calabasas Hills, CA, USA). The tests for insulin and glucagon have not been validated in dolphins. Fasting and 2 h post-prandial samples were 132–706 days (489 days on average) apart.

### Serum hematological analysis and calculations

Serum biochemistry and CBC analyses were performed at the Naval Medical Center using a Cobas 8000 Modular Analyzer Series (Roche Diagnostics, Indianapolis, IN, USA) and Beckman Coulter LH755 (Beckman Coulter, Brea, CA, USA), respectively, as well as at Quest Diagnostics using a Coulter LH 750 (Beckman Coulter, Fullerton, CA, USA) and Olympus AU600 (Olympus America, Center Valley, PA, USA). Serum chemistry analyses included glucose, sodium, potassium, chloride, carbon dioxide, anion gap, creatinine, blood urea nitrogen (BUN), uric acid, total bilirubin, total protein, alkaline phosphatase (ALP), ALT, AST, lactate dehydrogenase (LDH), gamma-glutamyl transpeptidase (GGT), and iron. CBC analyses included total white blood cell count with differential, red blood cell count, hemoglobin, hematocrit, mean corpuscular volume, mean corpuscular hemoglobin, and mean corpuscular hemoglobin concentration (MCHC). EDTA plasma samples correlating to serum samples used were analyzed by Esoterix Inc., Laboratory Services for total insulin and glucagon. Insulin was measured by two-site manual immunochemiluminometric assay (ICMA). Glucagon was measured by radioimmunoassay (RIA) following addition of aprotinin injection (Trasylol^®^, Bayer Pharmaceuticals Corporation, West Haven, CT, USA).

Homeostasis Model Assessment for Insulin Resistance (HOMA-IR) was calculated using a standard equation used in human endocrinology: [plasma insulin (μIU/ml) × serum glucose (mg/dl)]/405 (24). Erythrocyte sedimentation rates (ESR; 60-min) were analyzed in-house from EDTA whole blood using Fisherbrand Dispette 2^®^(Fisher Scientific, Waltham, MA, USA), correlating with the Westergren method. Estimated glomerular filtration rate (eGFR) was calculated using a dolphin-specific equation based on serum creatinine (25).

### Reagents and chemicals

All reagents were ACS grade or higher. Water and acetonitrile were LC-MS grade (Honeywell Burdick and Jackson, Morristown, NJ, USA). Solutions of ammonium bicarbonate, dithiothreitol (DTT), and iodoacetamide (IAA) were made fresh each day. Only Protein Lo-Bind (Eppendorf, Hamburg, Germany) microcentrifuge tubes were used to minimize protein and peptide adsorption.

### Depleted serum preparation for target peptide selection

To confirm target proteotypic adiponectin peptides, an aliquot of the “method development serum” was utilized. Protein concentrations were determined by the Bio-Rad Protein assay (Hercules, CA, USA) which is based on the Bradford method. A Multiple Affinity Removal System (MARS) Human 14 spin cartridge (Agilent Technologies, Santa Clara, CA, USA) was used to deplete 30 μl of serum (3 × 10 μl) which depleted the sample from 1000 to 139 μg protein. The resulting eluent was precipitated with acetone, then reconstituted in *Rapi*Gest SF Surfactant (Waters, Milford, MA, USA), reduced with 5 mM DTT, alkylated with 15 mM IAA, and digested overnight with Promega Gold trypsin (Promega, Madison, WI, USA) at an enzyme-to-protein ratio of 1:50 (w:w). The digest was halted with 0.1% formic acid (v/v) and the resulting peptides were desalted using a 30 mg Strata-X 33 μ polymeric reverse phase solid phase extraction column (Phenomenex, Torrance, CA, USA), washed with 1 ml 5% acetonitrile in 0.1% formic acid, and eluted with 1 ml 60% acetonitrile in 0.1% formic acid. The eluent was dried under vacuum with spinning followed by resuspension in mobile phase A (MPA) (98% water, 2% acetonitrile, 0.1% formic acid) to a concentration of 0.25 μg/μl protein (as estimated by Abs_280_), and 10 μl was injected onto the LC-MS/MS as described below.

### Synthetic peptide standards

The following peptides were synthesized by New England Peptide (Gardner, MA, USA) with isotopically labeled (“^∧^”; ^13^C and ^15^N) c-terminus amino acids: IFYNQQSHYDGTTGK^∧^ (IFY) and GDTGETGVTGVEGPR^∧^ (GDT). The manufacturer verified labeled peptide concentration by amino acid analysis of an aliquot made from 0.5 mg of each standard, after which 100 μl aliquots at approximately 200 μM in 30% acetonitrile (v/v) 0.1% formic acid (v/v) were frozen at −80°C. For collision energy optimizations, external calibration curves, and sample analyses, fresh aliquots were used by first thawing at 37°C for 1 min and then diluted in 10% acetonitrile (v/v) 0.1% formic acid (v/v) to achieve the desired concentration.

### Preparation of tryptic peptides for analysis

Prior to digesting the samples, a protocol was optimized for digesting dolphin serum. The amount of serum, acid-cleavable detergent, trypsin, and different desalting elutions were evaluated to maximize assay sensitivity and eliminate sources of experimental error. The resulting protocol is as follows: frozen serum aliquots were thawed at 37°C for 1 min and vortexed for 5 s. A 5 μl aliquot of serum was transferred to a 1.5 ml microcentrifuge tube, and 45 μl 50 mM ammonium bicarbonate (AmBic) was added. To this mixture, 50 μl of 0.1% (w/v) Progenta™ Anionic Acid Labile Surfactant II (AALS2; Progenta™ Protea Biosciences, Morgantown, WV, USA) dissolved in 50 mM AmBic was added and mixed by pipetting. To this mixture 11 μl of 1 M DTT (in 25 mM AmBic) was added and incubated for 30 min at 60°C. The reaction was allowed to cool 5 min before 12.5 μl IAA (in 100 mM AmBic) was added and incubated in the dark for 30 min at 37°C. The reaction was brought to 200 μl with 50 mM AmBic before trypsin was added at an enzyme-to-protein ratio of 1:10 (w:w). Finally 100 μl 100 mM AmBic was added and the digest was incubated for 16 h at 37°C in the dark. After 16 h, the digest was stopped with the addition of 350 μl 1% formic acid (v/v), and left at room temperature for 30 min to allow for the AALS2 to be cleaved. Next, 300 μl of 0.1% formic acid was added to bring the final volume to approximately 1 ml. Additionally, 30 μl of a 100 fmol/μl internal standard (IS) mix comprised of the two isotopically labeled peptides in 10% acetonitrile (v/v) was added, which corresponded to 3000 fmol of each IS. Following the manufacturer’s protocol, the complete volume was loaded onto a methanol-conditioned 30 mg Strata-X 33 μ polymeric reverse phase solid phase extraction column (Phenomenex, Torrance, CA, USA) and washed three times with 0.1% formic acid followed by 5% acetonitrile (v/v) in 0.1% formic acid. The peptides were eluted using 1 ml 15% acetonitrile in 0.1% formic acid. The elution was frozen at −80°C, and dried under vacuum by speedvac. Next 100 μl of MPA (98% water, 2% acetonitrile, 0.1% formic acid) was added, the sample was vortexed 15 min, centrifuged at 10,000 × *g* for 5 min, and the resulting supernatant was transferred to a clean 1.5 ml microcentrifuge tube. At this stage, the sample protein concentration was approximately 7 μg/μl (as estimated by Abs_280_), therefore prior to injection, 25 μl was diluted into 975 μl of MPA, and was analyzed by LC-MS/MS.

### LC-MS/MS

Prior to analysis by tandem mass spectrometry, the performance of the LC-MS/MS was confirmed using a standard tryptic digest of β-galactosidase by evaluating precursor retention times, and fragment ion areas. Tryptic peptides (10 μl) were injected onto a 100 μm × 1 cm C18 (100 Å with 5-mm particles) trap column (Acclaim PepMap 100; Thermo Fisher Scientific), and separated on a 75 μm × 15 cm C18 (300 Å with 3-μm particles) analytical column (Acclaim PepMap 100; Thermo Fisher Scientific). Reverse phase separation at 300 nl/min was performed with a gradient of 3–30% mobile phase B [95% acetonitrile (v/v), 0.1% formic acid (v/v)] over 12 min on a 2D+ NanoLC system (Eksigent, Dublin, CA, USA). The LC was interfaced to a TripleTOF 5600 System (AB Sciex, Foster City, CA, USA) with a nanospray source. Source temp was set at 120°C, and source voltage was set at 2500 V. The declustering potential was set at 110 V. To identify target proteotypic peptides, the instrument was run in positive ion instrument dependent acquisition mode with precursor ion scans for 250 ms with up to 20 product ion scans of 50 ms if precursors were 300–1250 *m/z*, exceeded 125 cps, and had a 2+ to 5+ charge state. For PRM experiments, the dominant charge state of each peptide was chosen and collision energies were optimized for each peptide. In one case the 2+ precursor was used, GDT, while the 3+ precursor was used for IFY. Collision energies were optimized for each peptide by monitoring the relative intensity of abundant fragment ions that had an *m/z* greater than the precursor *m/z*. The value which yielded the highest fragment ion area was used, which typically was close to the CE predicted by the equation determined by Kuzyk et al. (26): CE = 0.043 × (precursor ion *m/z*) + 2.25. For PRM, the instrument was set in positive ion mode and collected TOF-MS data in a window of 450–1250 *m/z* for 150 ms, followed by each parent ion MS/MS for 400 ms, collecting data from 100 to 1600 *m/z*. Due to the nature of the TripleTOF mass spectrometer, all ion pairs were monitored. The precursor masses were cycled such that the unlabeled peptide was collected, followed by the corresponding isotopically labeled peptide, before moving to the next peptide. For each peptide the three fragment ions with highest abundance were selected and used for PRM experiments (Figure S1 and Table S1 in Supplementary Material).

### Peptide identification

For determination of target peptides, raw data generated by the AB Sciex 5600 were converted to a peak list using the AB Sciex MS Data Converter (v. 1.1 beta, July 2011). Protein identifications were made using Mascot (v. 2.3.02) searching against the Ensembl (release 64) turTru1 dolphin genome assembly protein database [16,598 sequences; (27)] and the common Repository of Adventitious Proteins database (cRAP; 2012.01.01; the Global Proteome Machine) using the following parameters: trypsin was selected as the enzyme and three missed cleavages were allowed; carbamidomethylation (Cys) was specified as a fixed modification; glucosylgalactosyl (Lys), oxidation (Met, Pro), and 2-succinyl (Cys) were specified as variable modifications; a precursor tolerance of 10 ppm and fragment ion tolerance of 0.5 Da; instrument type was ESI-QUAD-TOF. The mass spectrometry proteomics data have been deposited to the ProteomeXchange Consortium (http://proteomecentral.proteomexchange.org) via the PRIDE partner repository (28) with the dataset identifier PXD000431.

### External calibration curves and adiponectin measurement

An external calibration curve was constructed using a mix of both labeled peptides in a digestion matrix with a peptide concentration approximately the same as the test samples. This was accomplished by digesting and processing the “method development serum” sample using the optimized protocol. After SPE clean up, the digest was brought up in 100 μl MPA, and 5 μl of this was diluted into 175 μl MPA and 20 μl of a 10× standard solution (for each calibration point) resulting in approximately 0.5 μg/μl protein digest. Twelve concentrations of labeled peptide were used from 0.005 to 10 fmol/μl, and 10 μl of each was injected onto the LC-MS/MS in triplicate and the instrument acquired data as described above. Quantitative measurements were calculated by using area under the curve for ion pairs using the built-in MQ4 algorithm in MultiQuant (v 2.0.2, AB Sciex). The following parameters were used for peak determination: 3.0 Gaussian smooth width, minimum peak width of three points, minimum peak height of 0.00. Integration parameters were as follows: 40% noise, 2 min baseline subtraction window, four points peak splitting. Fragment ions were extracted from each MS/MS experiment using a predicted monoisotopic mass ± 0.05 *m/z*. Regression was performed using peak areas with a linear fit and a *1/x* weighting. There was a linear relationship of instrument response to analyte concentration from 0.05 to 100 fmol for all peptides and relevant fragment ions (Figures S2–S7 in Supplementary Material). Using the “best” ion pair for each peptide (IFY y13^2+^ and GDT y7), there was no concentration that had relative standard deviations higher than 20%; therefore experimental assay variability and limits of detection and quantification were determined within experimental analysis as opposed to using the external curve for such calculations. An experimental blank was processed during each digestion batch, which was 5 μl phosphate buffered saline digested exactly the same as test samples, with 15 μg trypsin used for digestion. This blank was used to calculate the limit of detection (LOD) and limit of quantification (LOQ) on the days of analysis. This was accomplished by measuring the standard deviation of triplicate blank area measurements and the following equations: (*m* = slope of external calibration curve) LOD = 3 σ/m; LOQ = 10 σ/m (29). Additionally, one sample was digested in triplicate during each batch of digestion and was analyzed to determine experimental variability. Lastly, a composite serum sample was used as an in-house internal reference material to correct between experimental batches. The peak areas for the y13^2+^ ion of IFY and the y7 ion of GDT of both the labeled and unlabeled peptides were extracted with MultiQuant and used to calculate the amount of adiponectin in samples. Experimental variability was calculated using experimental triplicates and was <15% RSD (Table S3 in Supplementary Material). Daily LOD and LOQ were calculated for the assay and the serum measurements were more than two orders of magnitude above LOQ. Additionally, the IS area and the average analyte area were within one order of magnitude. Taken together, these parameters indicated the assay was performing with high sensitivity and precision. Lastly, the measurement of IFY was used to indicate total adiponectin and the measurement of GDT to indicate the amount of unmodified Lys75 adiponectin such that percent unmodified Lys75 adiponectin is: (GDT/IFY) × 100.

### Statistics

Wilcoxon rank-sum test was used to compare continuous variables between two groups with both SAS (v 9.1, SAS Incorporated) and MATLAB (R2013a; Mathworks), and since there were fewer than 10 individuals per category, the exact test was performed. Fisher’s exact test was used to compare categorical factors. Pearson Product Moment Correlation analysis was performed with SigmaPlot 11.0 (Systat Software).

## Results

### Development of adiponectin assay

**Figure 1:**
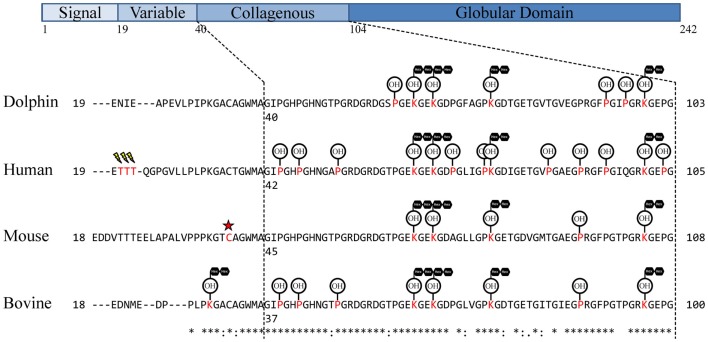
**Observed dolphin adiponectin post-translational modifications**. Adiponectin residues were aligned using UniProt alignment (ClustalO) with UniProt/SwissProt sequences and the Ensembl *Tursiops truncatus* sequence. Signal peptides were removed and sequences manually aligned around Human Thr20–Thr22 similar to Richards et al. (35). The collagen domain was assigned based on G-X-Y-G repeats as in Wang et al. (33). Assigned modifications for comparison are from previous studies of adiponectin in human (32, 35), mouse (30, 34, 37), and cow (31). Modified residues are in red, sialylation is represented as a lightning bolt, succination represented as a red star, hydroxylation represented as OH encircled, glycosylation [specifically, glucosyl-α(1,2)galactosylation] represented as black hexagons in tandem.

In order to select proteotypic adiponectin peptides for quantification by PRM, digested endogenous dolphin adiponectin was analyzed by tandem mass spectrometry. Using a *Tursiops truncatus* protein database and by specifying known and predicted adiponectin modifications (30–36) nine peptides were identified for a total of 43% coverage of the 242 amino acid dolphin adiponectin (Ensemble ID ENSTTRP00000015964; Table 1). Three hydroxylated prolines (Pro60, Pro93, Pro96) and four glucosylgalactosyl lysines (Lys63, Lys66, Lys75, Lys99) in the collagenous domain were identified (Figure 1). There was no evidence of succination (Cys). From the list of identified peptides and corresponding predicted chemical properties (e.g., solubility) two target peptides were selected: IFYNQQSHYDGTTGK (IFY) and GDTGETGVTGVEGPR (GDT). Sample processing and instrument parameters were optimized to measure these two peptides in dolphin serum using PRM.

**Table 1:** **Identified adiponectin peptides. and their modifications**.

Position	Peptide sequence and modifications	Ion score	Expect value
57–90	DGS**P**GE**K**GE**K**GDPGFAGP**K**GDTGETGVTGVEGPR	70	1.3E–6
	*3 Glucosylgalactosyl (K); Oxidation (P)*	
64–90	GE**K**GDPGFAGP**K**GDTGETGVTGVEGPR	41	6.7E–4
	*2 Glucosylgalactosyl (K)*	
76–90	GDTGETGVTGVEGPR	110	2.6E–5
91–98	GF**P**GI**P**GR	30	0.002
	*2 Oxidation (P)*	
99–110	**K**GEPGESAYVYR	103	5.4E–10
	*Glucosylgalactosyl (K)*	
111–120	SAFSVGLETR	62	4.2E–6
121–129	VTIPNVPIR	53	1.0E–5
133–147	IFYNQQSHYDGTTGK	75	1.1E–6
191–205	NVDQASGSVLLYLEK	72	4.0E–7

Tryptic peptides from dolphin adiponectin were identified by tandem mass spectrometry followed by database searching using Mascot. Modified residues predicted by Mascot are in bold and underlined, with modifications indicated below each peptide sequence. Peptide position relative to the whole protein, Mascot ion scores, and statistical probability of the identity match being random (Expect Value) are presented.

### Iron overload case-control study

While comparisons are limited based upon the small sample size, there were no significant differences in gender, age, weight, length, BMI, or diet between the two groups (Table 2). Blood values were compared between cases and controls within the fasting and 2 h post-prandial sample sets (Table 3). Within the fasting samples, ALT and GGT were significantly higher in the iron overload group (*p* = 0.0159 and 0.0476, respectively) while MCHC was significantly lower (Table S2 in Supplementary Material; *p* = 0.0317) compared to controls. Iron was not significantly different, which was expected since the iron overload samples were taken post-phlebotomy (21). Within the post-prandial set, ALT was also higher and MCHC (Table S2 in Supplementary Material) was lower in the iron overload group (*p* = 0.0159 and 0.0159, respectively) compared to controls. Additionally, total bilirubin, anion gap (Table S2 in Supplementary Material), insulin, and HOMA-IR were higher in the iron overload group (*p* = 0.0476, 0.0357, 0.0159, and 0.032, respectively) compared to controls. Specifically, insulin was 39.0 ± 18.8 μIU/ml and 7.7 ± 5.8 μIU/ml and HOMA-IR scores were 10 ± 5 and 2 ± 1, for iron overload cases and controls, respectively.

**Table 2:** **Study group characteristics table**.

Characteristic	Iron overload	Control	*p*-Value
**SEX, *N* (%)**			
Female	2 (50%)	4 (80%)	0.524
Male	2 (50%)	1 (20%)	
**AGE (years)**			
Mean ± SD	34.3 ± 7.5	26 ± 15.2	0.286
Median	32.4	26.9	
Range	27.5–44.8	8–47.9	
**WEIGHT (kg)**			
Mean ± SD	200.3 ± 45.3	186.1 ± 32.3	0.730
Median	196.8	172.3	
Range	160–247.7	147.7–223.2	
**LENGTH (cm)**			
Mean ± SD	261 ± 20	253 ± 8	0.857
Median	260	255	
Range	241–284	242–264	
**BMI (kg/m^2^)**			
Mean ± SD	29 ± 2.4	29 ± 4.5	0.556
Median	29.1	26.5	
Range	26.5–31.4	25.2–36	
**FED PROTEIN (g/kg)**			
Mean ± SD	6.3 ± 1	6.6 ± 1.4	0.556
Median	6.4	6.8	
Range	4.9–7.2	5.1–8.4	

All data is temporally related to the 2 h post-prandial sample set, and dietary data is from the draw date’s protein load. Comparisons between the groups were made using Fisher’s exact test for categorical factors and Wilcoxon rank-sum test for continuous variables.

**Table 3:** **Hematologic and serum biochemistry data of study group**.

	2 h post-prandial			Fasting		
	Iron overload	Control	*p*-Value	Iron overload	Control	*p*-Value
WBC (10^3^/μl)	8.5 ± 1.0	8.7 ± 3.4	0.730	10.2 ± 3.6	7.5 ± 1.1	0.191
HGB (g/dl)	12.6 ± 2.5	12.9 ± 0.8	0.730	12.0 ± 1.0	13.0 ± 0.8	0.214
Platelets (10^3^/μl)	57.3 ± 23.0	78.2 ± 18.5	0.191	78.8 ± 14.6	104.0 ± 36.1	0.286
Eosinophils (cells/μl)	746 ± 266	1025 ± 900	0.905	1024 ± 638	1218 ± 365	0.905
Glucose (mg/dl)	98 ± 9	102 ± 7	0.905	117 ± 33	109 ± 16	0.556
Protein (g/dl)	7.0 ± 0.4	6.4 ± 0.4	0.079	7.3 ± 0.2	6.6 ± 0.5	0.056
Albumin (g/dl)	4.2 ± 1.9	4.9 ± 0.3	0.960	5.0 ± 0.8	4.2 ± 0.3	0.214
Globulins (g/dl)	–	–	–	2.9 ± 0.1	2.4 ± 0.3	0.095
ALP (U/l)	290 ± 152	320 ± 100	0.730	259 ± 134	262 ± 104	0.905
LDH (U/l)	476 ± 106	455 ± 55	1.000	462 ± 147	346 ± 70	0.214
AST (U/l)	275 ± 49	190 ± 111	0.191	346 ± 97	229 ± 100	0.191
ALT (U/l)	40 ± 5	27 ± 6	0.016*	56 ± 9	29 ± 8	0.016*
GGT (U/l)	50 ± 18	29 ± 7	0.079	66 ± 35	30 ± 9	0.048*
Bilirubin (mg/dl)	0.2 ± 0.1	0.0 ± 0.1	0.048*	0.2 ± 0.1	0.1 ± 0.1	0.119
Cholesterol (mg/dl)	234 ± 92	203 ± 25	1.000	245 ± 71	199 ± 29	0.286
Triglycerides (mg/dl)	101 ± 10	101 ± 36	0.786	124 ± 86	71 ± 18	0.191
Fe (μg/dl)	205 ± 65	162 ± 65	0.413	210 ± 85	159 ± 29	0.516
ESR (mm/h)	8 ± 11	13 ± 6	0.413	15 ± 21	9 ± 6	0.849
eGFR (ml/min/2.78m^2^)	217 ± 30	271 ± 32	0.087	199 ± 8	243 ± 39	0.119
Insulin (μIU/ml)	39 ± 19	8 ± 6	0.016*	14 ± 12	17 ± 17	0.849
Glucagon (pg/ml)	155 ± 54	87 ± 54	0.286	–	–	–
HOMA-IR	10 ± 5	2 ± 1	0.032*	4 ± 4	5 ± 5	0.730

A Wilcoxon rank-sum test was used to compare blood chemistry values between groups, and significance indicated by “*” if *p* < 0.05. A complete table of hematologic and serum biochemistry values can be found in Table S2 in Supplementary Material.

**Figure 2:**
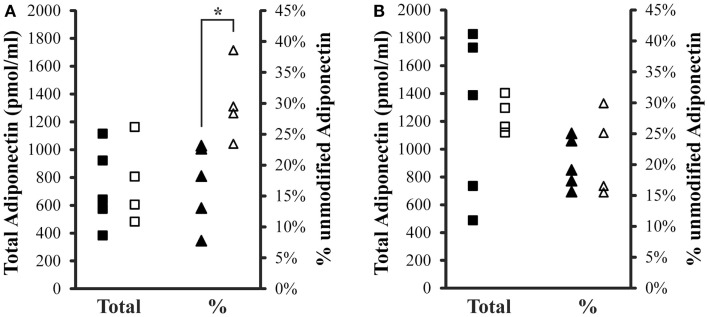
**Total and percent unmodified adiponectin levels in post-prandial and fasting samples**. **(A)** Two hours post-prandial and **(B)** fasting sample sets. Squares are total adiponectin and triangles are percent unmodified adiponectin. Solid symbols are controls and hollow symbols are iron overload cases. An “*” indicates significance difference (*p* < 0.05) when comparing the groups.

Total (IFY) and percent unmodified Lys75 adiponectin (GDT) were compared between controls and iron overload cases within fasting or post-prandial samples (Figure 2). Total adiponectin was not different between the two groups either fasting or post-prandial. The average total adiponectin (±SD) in the fasting samples was 1233 ± 597 pmol/ml and 1245 ± 130 pmol/ml for control and iron overload subjects, respectively. The average total adiponectin in the post-prandial samples was 727 ± 291 pmol/ml and 763 ± 298 pmol/ml for control and iron overload subjects, respectively. Percent unmodified adiponectin was not different between the two groups within the fasting samples at 20.2 ± 4.1 and 21.7 ± 6.9% in control and iron overload samples, respectively. Percent unmodified adiponectin was significantly higher in iron overload subjects within the post-prandial samples (*p* = 0.016) at 17.0 ± 6.6 and 30.0 ± 6.3% in control and iron overload subjects, respectively. Using a threshold of greater than 23.1% unmodified adiponectin, these two groups were completely separated in the post-prandial samples.

Levels of total and percent unmodified adiponectin were also compared to evaluate gender differences. In the fasting sample set, total adiponectin in males was 1362 ± 59 pmol/ml (with 26.7 ± 2.8% unmodified) and in females was 1177 ± 529 pmol/ml (with 18.0 ± 3.2% unmodified). In the 2 h post-prandial samples, total adiponectin in males was 1023 ± 194 pmol/ml (with 25.3 ± 3.6% unmodified) and in females was 601 ± 183 (with 21.4 ± 11.1% unmodified).

**Figure 3:**
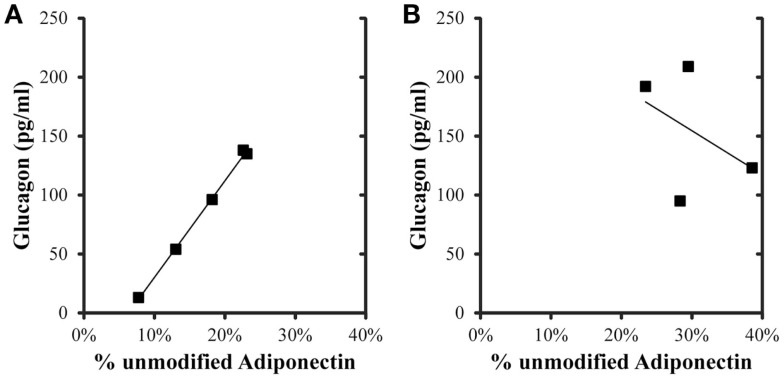
**Glucagon and percent unmodified adiponectin levels in the 2 h post-prandial samples**. The linear regression in **(A)** control group (*n* = 5) was *r* = 0.999, *p*  < 0.001, and in the **(B)** iron overload group (*n* = 4) was *r* = 0.432,*p* = 0.568.

Correlations were evaluated between metabolic variables, total adiponectin, and percent unmodified adiponectin in the post-prandial samples (Table 4). For the control group, total, and percent unmodified adiponectin were significantly positively correlated (*p* < 0.05) with each other. Both total and percent unmodified adiponectin were significantly positively correlated with glucagon levels (ρ = 0.934 and 0.999, respectively; *p* < 0.05; Figure 3). Neither were significantly correlated to glucose, insulin levels, or HOMA-IR. Comparing the same variables in the iron overload subjects, there was no significant correlation. Specifically, the striking correlation of glucagon and percent unmodified adiponectin in the controls (Figure 3A) was completely absent in the iron overload cases (Figure 3B).

**Table 4:** **Pearson product moment correlations (ρ) of variables within groups in the 2 h post-prandial sample set**.

	Unmodified adiponectin (%)	Glucose	Insulin	Glucagon	HOMA-IR
**CONTROL GROUP**					
Total adiponectin	ρ = 0.940	ρ = 0.160	ρ = 0.257	ρ = 0.934	ρ = 0.260
	*p* = 0.0174*	*p* = 0.797	*p* = 0.676	*p* = 0.0204*	*p* = 0.672
Unmodified adiponectin (%)	–	ρ = 0.136	ρ = 0.393	ρ = 0.999	ρ = 0.404
	–	*p* = 0.828	*p* = 0.512	*p* < 0.001*	*p* = 0.500
**IRON OVERLOAD GROUP**					
Total adiponectin	ρ = −0.245	ρ = 0.725	ρ = −0.370	ρ = 0.927	ρ = −0.266
	*p* = 0.755	*p* = 0.275	*p* = 0.630	*p* = 0.0727	*p* = 0.734
Unmodified adiponectin (%)	–	ρ = −0.485	ρ = −0.607	ρ = −0.432	ρ = −0.623
	–	*p* = 0.515	*p* = 0.393	*p* = 0.568	*p* = 0.377

Correlations between metabolic variables were evaluated within controls and iron overload cases. Significant correlations are indicated by “*”, *p*  < 0.05.

## Discussion

### Assay development for dolphin adiponectin

In this study tryptic peptides belonging to adiponectin from the bottlenose dolphin were identified using liquid chromatography tandem mass spectrometry. These peptides were used to develop a reaction monitoring assay to quantify adiponectin in dolphin serum. Since a high resolution mass spectrometer was used that did not isolate individual fragment ions in a distal quadrupole, this type of analysis has been termed PRM (16). Reaction monitoring is gaining popularity as a tool for estimating protein quantity using peptides derived from proteolytic digestion (14), with the added benefit that it can be multiplexed and provides a level of specificity through direct analyte detection that is not afforded by antibody-based techniques. As this study highlights, one of the more liberating aspects of reaction monitoring is the fact that it can be quickly adapted to measure clinically relevant proteins in non-model organisms if a protein sequence is known and proteolytic peptides can be detected. Moreover, this data demonstrates that a mid-abundance serum protein, adiponectin (0.06% of total serum protein in the method development sample), can be quantified without depletion or enrichment prior to analysis. Quantifying proteins in this direct manner has been demonstrated before using SRM (38–40).

The range of total adiponectin concentrations in the study population was 383–1827 pmol/ml, corresponding to 10–48 μg/ml adiponectin. This is within the reported range for humans (41), mice (42), dogs (43), and cows (44) (see Table S4 in Supplementary Material). In reference to reported adiponectin values from the elephant seal (9, 10), order Carnivora, the dolphin levels were approximately 20- to 170-fold higher, suggesting either a massive difference in the circulating level of adiponectin between marine mammals or a reflection of the assay utilized to measure adiponectin. Comparative generalizations made across mammals would be premature at this stage due to lack of standardization of assay methodology.

Adiponectin exists as a variety of multimers, dictated by PTMs in the collagenous domain. Oligomerization is key to directing the receptor-mediated biological activities of adiponectin. For instance, monomeric adiponectin produced by recombinant bacterial systems does not form HMW multimers compared to adiponectin produced by mammalian expression systems, and thus does not repress serum glucose (45, 46). Further, the amount of HMW adiponectin (18–30+ protomers) but not total adiponectin correlates to improved insulin sensitivity in mice and humans with type 2 diabetes mellitus (47). Although oligomer formation is dependent on disulfide bond formation at Cys34 (34), lysine, and proline modifications within the collagenous domain are also required [reviewed in Ref. (33)]. For example, the level of HMW adiponectin is directly related to the extent of which the four conserved lysine residues are modified (17), while proline hydroxylation likely promotes rapid folding and thermostability, like that for other collagen-like domains (48). Further, published mutagenesis studies of four conserved adiponectin lysine residues demonstrated a shift in production from HMW to LMW multimers (17, 32). Together this suggests that the biological activity of adiponectin is directly related to the modification of key residues.

### Post-translational modification and ratiometric measurement of dolphin adiponectin

A search of tandem mass spectra from dolphin serum tryptic peptides was performed to determine whether known lysine and proline modifications were present on dolphin adiponectin. Glucosylgalactosyl modification of four lysines, Lys63, Lys66, Lys75, and Lys99 (Figure 1; Table 1), were identified which are conserved across mammals, though there was no evidence for glucosylgalactosyl modification of Lys31 reported for bovine adiponectin (31). There was also hydroxylation at proline residues Pro60, Pro93, and Pro96. The identification of hydroxylation at Pro60 (but not Pro69) was supported by b- and y-ion series, although hydroxylation of the human equivalent of Pro69 (but not Pro60) has been described [human Pro71; (32)]. Overall, these results confirm dolphin adiponectin can be modified at key lysine and proline residues in the collagenous domain, though it is unclear exactly how the degree of modification affects oligomerization.

In this study, a new method to measure adiponectin was developed by measuring two quantotypic tryptic peptides, IFY and GDT, by PRM. To date, two other studies have used more than one peptide to quantify adiponectin by reaction monitoring. Homologs of IFY and GDT have been used to quantify human adiponectin (49), while more recently, GFPGIQGR as well as human IFY have been targeted for measurement (50). These peptides are located within the collagenous domain (GFPGIQGR and GDT) and globular domain (IFY; Figure 1). Using the human homologs of IFY and GDT as well as the same fragment ions as this study, Domanski et al. found IFY was threefold higher than GDT in a pooled sample of human plasma (49). Likewise IFY was on average fivefold higher than GDT in dolphin serum. Interestingly, the GDT homolog in humans contains an additional proline (human Pro86), and both prolines (human Pro86 and Pro 91) have been shown to be hydroxylated (32, 35). Because the GDT measurement was based on measuring the unmodified peptide, it would be affected by either proline hydroxylation. In the current study, oxidation of dolphin Pro89 was not identified. In addition to internal proline residues, GDT is surrounded by proline (32) and lysine (33) residues known to be modified. Modification of Lys75 is likely to prohibit cleavage by trypsin at this residue and therefore result in lower levels of GDT compared to IFY. This is also supported by the observation of two additional peptides with mis-cleavages after a modified Lys75 (Table 1). In the case of GFPGIQGR, it is expected that human Lys101 and Pro95 modifications may be limiting cleavage and measurement, respectively, resulting in the sixfold lower measurement of this unmodified peptide versus the GDT homolog in a pooled human plasma (50).

Since Lys75 modification is relevant to the multimerization of and formation of HMW adiponectin, percent unmodified adiponectin at Lys75 was determined using the ratio of GDT to IFY. The percent unmodified Lys75 adiponectin in the study population was between 8 and 39%, suggesting that 61 to 92% of the adiponectin is modified at Lys75. The percentage of modified adiponectin is similar to levels of HMW adiponectin reported for humans [30–50%; (51)], cats [50%; (52)], and dogs [80%; (43)]. For this reason the level of percent unmodified Lys75 appears to be a proxy of multimeric adiponectin, although further validation is necessary to determine the distribution of this modification across different multimers.

### Comparison of total adiponectin levels in dolphins with iron overload

The hypothesis was tested that total adiponectin levels in dolphins with iron overload and putative insulin resistance have lower adiponectin levels compared to control dolphins. Serum levels were measured at two different states; fasting and 2 h post-prandial, the later state being the one at which both insulin and glucagon were elevated in the animals with iron overload compared to control animals [see Ref. (3)]. To avoid misrepresentation, blood samples were not drawn on the same day, but rather at completely different subsequent periods of time as stated in the methods. There was no statistical difference between total adiponectin levels in dolphins with iron overload versus controls during fast or 2 h post-prandial; therefore this hypothesis was rejected. The absolute amount of modified adiponectin as a proxy of multimeric HMW adiponectin was calculated based on the unmodified Lys75 peptide (i.e., GDT). There was no significant difference between iron overload cases and control dolphins regardless of fasting or feeding (data not shown). Although low total adiponectin levels have been reported in patients with non-alcoholic fatty liver disease (53) and inversely associated with serum ferritin and transferrin (54), total adiponectin does not appear to correspond to insulin resistance promoted by iron overload in dolphins.

Of note, the effect of gender and age on the level of adiponectin may be confounding in a small study population such as the one utilized in this study. In humans total adiponectin levels are higher in females than males, and increases with age in both sexes [reviewed by Brochu-Gaudreau et al. (55)]. Although the gender differences were not significantly different between both groups, females comprised 80% of the control group and 50% of the case group. Incongruent to that reported for other mammals, males tended to have higher total adiponectin levels and percent unmodified adiponectin than did females when dolphins were grouped by gender alone and classified by fasting or 2 h post-prandial. Based on this data, the group with the higher percentage of females (control) could have biased the result such that the hypothesis was less likely to have been accepted. Due to a limited number of animals available for study, caution should be taken when extrapolating interpretation of results to the population level, but also highlights the need for a future study with a larger frequency matched population.

### Ratiometric measurement of adiponectin in dolphins with iron overload

Because a reduction in the fraction of HMW adiponectin occurs in response to insulin or glucose administration in mice (56) and because the ratio of HMW to total adiponectin is correlated to improved hepatic insulin sensitivity (47), the fraction of unmodified Lys75 (i.e., GDT) adiponectin was measured in dolphins with iron overload and an insulin resistant phenotype. Although this assay has not yet been validated as a direct measure of multimeric forms of adiponectin (e.g., HMW adiponectin), the fact that lysine hydroxylation is correlated with adiponectin multimerization led us to speculate that the direct measurement of unmodified Lys75 (GDT) peptide relative to the total adiponectin peptide (IFY) could serve as a proxy for probable multimerization. If this assumption is valid, then the ratio of unmodified Lys75 peptide (GDT) to total adiponectin peptide (IFY) should be elevated in dolphins with iron overload at 2 h post-prandial because these animals have elevated insulin levels. During fasting, both groups of dolphins were essentially equivalent in the percent unmodified adiponectin; however, at 2 h post-prandial the percent of unmodified adiponectin was significantly higher in the iron overload cases (Figure 2). The elevation in 2 h post-prandial percent unmodified adiponectin in iron overload cases corresponds well to the fractional response of HMW adiponectin following oral glucose load or intraperitoneal insulin load in mice (56). The increase in percent unmodified Lys75 (GDT) peptide relative to the total adiponectin peptide (IFY) at the 2 h post-prandial time point also corresponded well with the elevated levels of insulin and glucagon reported by Venn-Watson et al. (3). These results support the assertion that GDT:IFY may serve as a proxy for HMW adiponectin multimers, but further studies are needed to confirm this speculation.

### Correlation of adiponectin with glucagon

To further explore additional relationships between the total and ratiometric measurements of adiponectin, the Pearson product moment correlations between adiponectin measurements and select serum biochemistry values were calculated (Table 4). Interestingly, only glucagon levels correlated with the total and ratiometric measurements of adiponectin in the control group. When plotted against glucagon, the ratiometric measurement of adiponectin results in a nearly perfect linear relationship with glucagon levels in control dolphins; whereas, in dolphins with iron overload there was no correlation (Figure 3). The discriminatory value of this relationship as a classifier for metabolic insufficiency is very strong and could potentially be valuable as a marker of hepatic insulin improvement such as that reported for humans (47).

To the authors’ knowledge, there are no previous studies describing such a relationship between glucagon and adiponectin in healthy mammals. A weak correlation between hepatic arterial adiponectin and glucagon was previously reported for patients with liver cirrhosis (57) and the authors speculated that the reason was because clearance routes were similar for both hormones; however, there was no correlation between total adiponectin and glucagon in dolphins with iron overload where hepatic dysfunction is more prevalent (2). It is possible that in healthy mammals, serum glucagon and adiponectin multimer concentrations are tightly controlled through mechanisms of clearance and that the correlation is lost in dolphins with hepatic dysfunction where glucagon is unable to be efficiently cleared and/or only higher molecular weight adiponectin is selectively cleared. In pigs, the clearance of glucagon largely occurs through elimination via the kidney and in dogs the fraction of glucagon extracted by the liver is only approximately 20% (58) suggesting that renal clearance, not hepatic clearance is the main sink. In this study there was no difference in eGFR between dolphin groups indicating that impaired renal clearance is an unlikely reason for preferential clearance of adiponectin forms. Adiponectin is cleared by both the liver and kidneys in mice with a half-life of about 75 min (59). However, adiponectin clearance does not appear to be as important as production in determining total serum adiponectin. In an obese, hyperinsulinemic mouse model (*ob/ob*), circulating adiponectin levels are low despite the fact that clearance is reduced suggesting that adiponectin production is disproportionately depressed (59). Further, hyperinsulinemia is known to reduce the circulating amount of HMV adiponectin independent of clearance (19), which corresponds to the idea that impaired insulin-signaling is a major determinant of adiponectin level (33).

Another potential mechanism connecting both adiponectin and glucagon centers around the ability of fibroblast growth factor 21 (FGF21) to suppress glucagon (60) and stimulate adiponectin secretion (61). FGF21 is a primarily liver derived fasting hormone that has been shown to reduce HOMA-IR, triglyceride levels, and blood glucose in the *db/db* mice when administered exogenously (62). Although speculative, the lack of correlation between glucagon and adiponectin in iron-overloaded dolphins might be partially explained by an inability to elevate FGF21 levels or a resistance to FGF21 signaling leading to improperly elevated glucagon levels and reduction in modified adiponectin. Because the link between FGF21, adiponectin, glucagon, and insulin resistance is very strong, future studies should consider measurements of FGF21 in dolphins. Taken together with the fact that insulin is a negative regulator of glucagon, and that dolphins with iron overload have significantly higher HOMA-IR scores, the evidence suggest that the higher ratio of GDT to IFY is most likely an effect of lower secretion of modified adiponectin coupled with improper glucagon feedback control.

### Adiponectin as an anti-inflammatory

Adiponectin is known to have other roles in addition to insulin sensitization one of which being as an anti-inflammatory mediator. Because there was no intergroup difference in ESR (Table 3), which is an indirect marker of inflammation, the correlation between systemic inflammation and circulating adiponectin in dolphins was not evident in this study. However, a hallmark feature of liver disease due to iron overload in dolphins is the accumulation of iron primarily in Kuppfer cells (macrophages) of the liver (2). The globular domain of adiponectin has been shown to elevate IL-10 and heme oxygenase 1 (HO-1), and reduce TNFα expression in Kupffer cells stimulated with lipopolysaccharide (63, 64). Given that inactivation of Kupffer with gadolinium chloride prevents ethanol-induced fatty liver disease in rats (65), any reduction in the efficacy of adiponectin to promote IL-10 and reduce TNFα could promote the fatty liver phenotype afflicting dolphins with iron overload. Although the effect of extracellular iron has not been reported with regards to dynamics of adiponectin modification, high iron has been shown to reduce total adiponectin in adipocytes (8). If proper adiponectin modification is necessary to suppress inflammation due to activated Kupffer cells, then it is possible that the link between non-alcoholic fatty liver disease, hemochromatosis and percent unmodified adiponectin could underlie the metabolic disorders that ensue.

### Future questions and conclusion

A number of basic questions remain unanswered with regards to adiponectin in dolphins. Based on a search of the dolphin genome, genes for adiponectin receptors R1, R2, and T-cadherin are present. Therefore, it is likely that homologous receptors for signaling exist in dolphins, but whether tissue receptor expression parallels human and rodent models and whether cell signal transduction pathways are conserved across species, remains to be established. Procollagen-lysine, 2-oxoglutarate 5-dioxygenase 3 (a.k.a. lysyl hydroxylase 3; Ensembl ID: ENSTTRG00000004781) is also present suggesting that differential enzyme activity responsible for lysine hydroxylation and subsequent glucosylgalactosylation of adiponectin in mice could underlie differences in the ratio of GDT to IFY (37). Whether or not adipocytes are the major source of adiponectin as in other mammals (33), remains to be determined.

In conclusion, an assay for adiponectin in dolphins has been established that relies on the highly specific measurement of tryptic peptides of adiponectin by PRM. Using this assay to study a small group of dolphins with iron overload has provided further insight into the hormonal dysregulation present in these animals. Moreover, these results highlight that a differential ratiometric measurement using two quantotypic peptides may serve as a marker of metabolic perturbations or improvement in dolphins. Future studies will address whether these peptide ratios can be used to determine the level of adiponectin modification and the relationship to degree of oligomerization. By defining a more precise adiponectin oligomer distribution along a continuum, more correlations with study endpoints could be evaluated. Because of the high sequence similarity between human and dolphin adiponectin and the presence of homologous gene products within both species, these findings may be directly applicable to the mechanistic understanding of both dolphin and human metabolism.

## Authors Contribution

Benjamin A. Neely contributed to the development of the assay, to the experimental design, carried out analysis on the mass spectrometer, handled data processing, and drafted the manuscript. Kevin P. Carlin contributed to the experimental design, gathering sample data, data processing, and assisted in drafting the manuscript. John M. Arthur assisted in the interpretation of mass spectrometry data, critical analysis of the assay design, and drafting the manuscript. Wayne E. McFee procured the original serum sample for the development of the assay and assisted in the writing and review of the manuscript. Michael G. Janech conceived of the assay and study, contributed to the development of the assay, to the experimental design, and assisted in drafting the manuscript. All authors read and approved the final manuscript.

## Conflict of Interest Statement

The authors declare that the research was conducted in the absence of any commercial or financial relationships that could be construed as a potential conflict of interest.

## Supplementary Material

The Supplementary Material for this article can be found online at http://www.frontiersin.org/Diabetes/10.3389/fendo.2013.00132/abstract

Figure S1 **Fragmentation tables of three synthetic isotopically labeled target peptides along with observed fragment ions**. Tables of fragment ion *m/z* is shown for each peptide (generated using the Institute for Systems Biology online Fragment Ion Calculator; http://db.systemsbiology.net/proteomicsToolkit/index.html). Observed fragment ions with highest peak intensities are labeled in MS/MS spectra, and colored red in tables.

Figure S2 **External calibration curves for yl3^2+^ from IFYNQQSHYDGTTGK**. The solid line represents the *1/x* weighted linear regression, *R* = 0.99181. Inset is provided to show performance at lower concentrations.

Figure S3 **External calibration curves for yl4^2+^ from IFYNQQSHYDGTTGK**. The solid line represents the *1/x* weighted linear regression, *R* = 0.98945. Inset is provided to show performance at lower concentrations.

Figure S4 **External calibration curves for yl2^2+^ from IFYNQQSHYDGTTGK**. The solid line represents the *1/x* weighted linear regression, *R* = 0.99056. Inset is provided to show performance at lower concentrations.

Figure S5 **External calibration curves for y7 from GDTGETGVTGVEGPR**. The solid line represents the *1/x* weighted linear regression, *R* = 0.99278. Inset is provided to show performance at lower concentrations.

Figure S6 **External calibration curves for y9 from GDTGETGVTGVEGPR**. The solid line represents the *1/x* weighted linear regression, *R* = 0.9873. Inset is provided to show performance at lower concentrations.

Figure S7 **External calibration curves for yl0 from GDTGETGVTGVEGPR**. The solid line represents the *1/x* weighted linear regression, *R* = 0.98691. Inset is provided to show performance at lower concentrations.

Table S1 **Ion pairs and instrument parameters used for PRM**. Each endogenous target peptide had an isotopically labeled synthetic version constructed with either a C-terminal 8Lys or 10Arg, designated by a “^∧^” and referred to as heavy. Fragment ion peak areas were extracted from each MS/MS experiment using these monoisotopic masses ± 0.05 *m/z*.

Table S2 **Complete hematologic and serum biochemistry data of study group**. A Wilcoxon rank-sum test was used to compare blood chemistry values between groups.

Table S3 **Assay performance measures**. To evaluate assay performance on the days of analysis, experimental triplicates were used quantify assay variability and detection limits. The average peak area ratio was determined based on all samples analyzed.

Table S4 **Measured adiponectin concentrations in blood from different organisms using different techniques**. Reported adiponectin measurements by ELISA are typically microgram per milliliter therefore concentrations were converted using the molecular weight of adiponectin from each species (an elephant seal was assumed to be exactly 26 kDa).
