# Supplementary material for: Ratiometric Measurements of Adiponectin by Mass Spectrometry in Bottlenose Dolphins (Tursiops truncatus) with Iron Overload Reveal an Association with Insulin Resistance and Glucagon
Source: Front Endocrinol (Lausanne). 2013 Sep 20;4:132. doi: 10.3389/fendo.2013.00132 (PMC3778387; doi:10.3389/fendo.2013.00132)
Supplement: Figure S1 — Fragmentation tables of three synthetic isotopically labeled target peptides along with observed fragment ions. Tables of fragment ion m/z is shown for each peptide (generated using the Institute for Systems Biology online Fragment Ion Calculator; http://db.systemsbiology.net/proteomicsToolkit/index.html). Observed fragment ions with highest peak intensities are labeled in MS/MS spectra, and colored red in tables. [file 65176_Janech_DataSheet1.ZIP › 65176_Janech_Table_S1.pdf]

Table S1. Ion pairs and instrument parameters used for PRM. Each endogenous target peptide had an isotopically labeled synthetic version constructed with either a C-terminal ^{8}Lys or ^{10}Arg, designated by a '^' and referred to as heavy. Fragment ion peak areas were extracted from each MS/MS experiment using these monoisotopic masses ± 0.05 *m/z*.

**Peptide**	**Label**	**Precursor *m/z***	**CE**	**y13^{2+}**	**y14^{2+}**	**y12^{2+}**
IFYNQQSHYDGTTGK	light	586.9	27	749.83	823.37	668.30
IFYNQQSHYDGTTGK^	heavy	589.6	27	753.83	827.37	672.30
				**y7**	**y9**	**y10**
GDTGETGVTGVEGPR	light	716.34	35	715.37	871.46	972.51
GDTGETGVTGVEGPR^	heavy	721.34	35	725.37	881.46	982.51
