# Supplementary material for: Ratiometric Measurements of Adiponectin by Mass Spectrometry in Bottlenose Dolphins (Tursiops truncatus) with Iron Overload Reveal an Association with Insulin Resistance and Glucagon
Source: Front Endocrinol (Lausanne). 2013 Sep 20;4:132. doi: 10.3389/fendo.2013.00132 (PMC3778387; doi:10.3389/fendo.2013.00132)
Supplement: Figure S1 — Fragmentation tables of three synthetic isotopically labeled target peptides along with observed fragment ions. Tables of fragment ion m/z is shown for each peptide (generated using the Institute for Systems Biology online Fragment Ion Calculator; http://db.systemsbiology.net/proteomicsToolkit/index.html). Observed fragment ions with highest peak intensities are labeled in MS/MS spectra, and colored red in tables. [file 65176_Janech_DataSheet1.ZIP › 65176_Janech_Table_S2.pdf]

Table S2. Complete hematologic and serum biochemistry data of study group. A Wilcoxon rank-sum test was used to compare blood chemistry values between groups.

	2 h post-prandial			Fasting		
	Iron overload	Control	*p* -value	Iron overload	Control	*p* -value
n	4	5		4	5	
WBC (cells/µl)	8,475 ± 991	8,660 ± 3,419	0.730	10,225 ± 3,624	7,500 ± 1,102	0.191
RBC (x10^6/µl)	2.95 ± 0.72	2.98 ± 0.14	1.000	2.90 ± 0.41	2.97 ± 0.21	1.000
HGB (g/dl)	12.6 ± 2.5	12.9 ± 0.8	0.730	12.0 ± 1.0	13.0 ± 0.8	0.214
HCT (%)	37.7 ± 6.7	37.6 ± 2.1	0.730	36.0 ± 2.4	37.3 ± 2.4	0.556
MCV (fl)	130.0 ± 16.7	126.3 ± 6.0	0.413	125.3 ± 11.3	126.0 ± 6.7	0.905
MCH (pg)	43.2 ± 5.0	43.2 ± 2.3	0.556	41.9 ± 3.9	44.0 ± 2.3	0.482
MCHC g/dl)	33.3 ± 0.9	34.3 ± 0.3	0.016*	33.4 ± 0.9	34.9 ± 0.6	0.032*
RDW (%)	17.0 ± 2.2	14.8 ± 0.8	0.191	16.2 ± 2.6	14.9 ± 0.8	0.286
NRBC (x10^6/µl)	1 ± 2	0 ± 0	0.444	0 ± 1	2 ± 4	1.000
Platelets (cells/µl)	57,250 ± 23,013	78,200 ± 18,499	0.191	78,750 ± 14,637	104,000 ± 36,125	0.286
MPV (fl)	13.8 ± 1.5	14.1 ± 1.8	0.778	14.0 ± 1.2	13.1 ± 1.2	0.127
Neutrophils (cells/µl)	5,715 ± 2,313	6,403 ± 2,367	0.905	6,229 ± 1,081	4,812 ± 725	0.111
Lymphocytes (cells/µl)	1,758 ± 1,304	1,134 ± 645	0.413	2,635 ± 3,453	1,374 ± 1,323	0.556
Monocytes (cells/µl)	249 ± 138	98 ± 136	0.183	335 ± 179	94 ± 132	0.064
Eosinophils (cells/µl)	746 ± 266	1025 ± 900	0.905	1024 ± 638	1218 ± 365	0.905
Glucose (mg/dl)	98 ± 9	102 ± 7	0.905	117 ± 33	109 ± 16	0.556
BUN (mg/dl)	51 ± 6	44 ± 3	0.079	54 ± 16	51 ± 5	0.857
Creatinine (mg/dl)	1.3 ± 0.2	1.1 ± 0.1	0.087	1.4 ± 0.1	1.2 ± 0.2	0.119
BUN:Creatinine	38 ± 7	40 ± 6	0.730	38 ± 11	42 ± 9	0.556
Uric acid (mg/dl)	0.6 ± 0.2	0.6 ± 0.2	0.532	0.2 ± 0.2	0.2 ± 0.2	0.968
Sodium (mEq/L)	154 ± 5	155 ± 1	0.413	153 ± 2	156 ± 2	0.111
Potassium (mEq/L)	3.9 ± 0.2	3.8 ± 0.1	0.810	3.6 ± 0.1	3.6 ± 0.5	0.841
Chloride (mEq/L)	116 ± 4	120 ± 2	0.206	120 ± 1	121 ± 2	0.452
CO2 (mEq/L)	25 ± 3	26 ± 2	0.548	24 ± 1	24 ± 2	0.587
Protein (g/dl)	7.0 ± 0.4	6.4 ± 0.4	0.079	7.3 ± 0.2	6.6 ± 0.5	0.056
Albumin (g/dl)	4.2 ± 1.9	4.9 ± 0.3	0.960	5.0 ± 0.8	4.2 ± 0.3	0.214
Globulins (g/dl)	(n=0)	(n=0)		2.9 ± 0.1 (n=2)	2.4 ± 0.3	0.095
Albumin:Globulin	(n=0)	(n=0)		1.5 ± 0.1 (n=2)	1.8 ± 0.2	0.238
Calcium (mg/dl)	9.0 ± 0.6	8.7 ± 0.3	0.587	9.1 ± 0.4	8.8 ± 0.2	0.452

Inorganic phosphate (mg/dl)	4.3 ± 0.2	4.2 ± 0.3	0.556	4.6 ± 0.8	5.1 ± 0.5	0.206
Alkaline phosphatase (U/L)	290 ± 152	320 ± 100	0.730	259 ± 134	262 ± 104	0.905
LDH (U/L)	476 ± 106	455 ± 55	1.000	462 ± 147	346 ± 70	0.214
AST (U/L)	275 ± 49	190 ± 111	0.191	346 ± 97	229 ± 100	0.191
ALT (U/L)	40 ± 5	27 ± 6	0.016*	56 ± 9	29 ± 8	0.016*
GGT (U/L)	50 ± 18	29 ± 7	0.079	66 ± 35	30 ± 9	0.048*
Bilirubin (mg/dl)	0.2 ± 0.1	0.0 ± 0.1	0.048*	0.2 ± 0.1	0.1 ± 0.1	0.119
Cholesterol, total (mg/dl)	234 ± 92 (n=3)	203 ± 25	1.000	245 ± 71	199 ± 29	0.286
Triglycerides (mg/dl)	101 ± 10 (n=3)	101 ± 36	0.786	124 ± 86	71 ± 18	0.191
Iron (µg/dl)	205 ± 65	162 ± 65	0.413	210 ± 85	159 ± 29	0.516
CPK (U/L)	124 ± 49	162 ± 65	0.413	111 ± 20	122 ± 63	1.000
ESR (mm/h)	8 ± 11	13 ± 6	0.413	15 ± 21	9 ± 6	0.849
Magnesium	2.1 ± 0.3 (n=3)	2.0 ± 0.1	0.643	2.3 ± 0.1 (n=3)	2.0 ± 0.0 (n=2)	0.200
Anion gap	14 ± 2 (n=3)	9 ± 1	0.036*	11 ± 1 (n=2)	(n=0)	
eGFR	217 ± 30	271 ± 32	0.087	199 ± 8	243 ± 39	0.119
HOMA-IR	10 ± 5	2 ± 1	0.032*	4 ± 4	5 ± 5	0.730
Insulin, total (uIU/ml)	39 ± 19	8 ± 6	0.016*	14 ± 12	17 ± 17	0.849
Glucagon (pg/ml)	155 ± 54	87 ± 54	0.286			

* Significant p-value (< 0.05)
