# Supplementary material for: Ratiometric Measurements of Adiponectin by Mass Spectrometry in Bottlenose Dolphins (Tursiops truncatus) with Iron Overload Reveal an Association with Insulin Resistance and Glucagon
Source: Front Endocrinol (Lausanne). 2013 Sep 20;4:132. doi: 10.3389/fendo.2013.00132 (PMC3778387; doi:10.3389/fendo.2013.00132)
Supplement: Figure S1 — Fragmentation tables of three synthetic isotopically labeled target peptides along with observed fragment ions. Tables of fragment ion m/z is shown for each peptide (generated using the Institute for Systems Biology online Fragment Ion Calculator; http://db.systemsbiology.net/proteomicsToolkit/index.html). Observed fragment ions with highest peak intensities are labeled in MS/MS spectra, and colored red in tables. [file 65176_Janech_DataSheet1.ZIP › 65176_Janech_Table_S3.pdf]

Table S3. Assay performance measures. To evaluate assay performance on the days of analysis, experimental triplicates were used quantify assay variability and detection limits. The average peak area ratio was determined based on all samples analyzed.

Peptide	Fragment Ion	Average peak area ratio (A:IS)[a]	Analyte Response (slope)[b]	Correlation coefficient (*r*)	fold range	LOD[c]	LOQ[c]	%RSD[d]
IFYNQQSHYDGTTGK	$y13^{2+}$	1.370	10703.032	0.99181	2000	1.62	5.41	7.2
GDTGETGVTGVEGPR	y7	0.326	9565.510	0.99278	2000	0.26	0.86	12.0

[a] area of analyte to area of internal standard

[b] fmoles on column versus area

[c] pmol/ml serum (the highest experimental LOD and LOQ are given)

[d] average % Relative Standard Deviation determined from measurements of two experimental triplicates
