# Supplementary material for: Ratiometric Measurements of Adiponectin by Mass Spectrometry in Bottlenose Dolphins (Tursiops truncatus) with Iron Overload Reveal an Association with Insulin Resistance and Glucagon
Source: Front Endocrinol (Lausanne). 2013 Sep 20;4:132. doi: 10.3389/fendo.2013.00132 (PMC3778387; doi:10.3389/fendo.2013.00132)
Supplement: Figure S1 — Fragmentation tables of three synthetic isotopically labeled target peptides along with observed fragment ions. Tables of fragment ion m/z is shown for each peptide (generated using the Institute for Systems Biology online Fragment Ion Calculator; http://db.systemsbiology.net/proteomicsToolkit/index.html). Observed fragment ions with highest peak intensities are labeled in MS/MS spectra, and colored red in tables. [file 65176_Janech_DataSheet1.ZIP › 65176_Janech_Table_S4.pdf]

**Table S4. Measured adiponectin concentrations in blood from different organisms using different techniques.** Reported adiponectin measurements by ELISA are typically µg/ml therefore concentrations were converted using the molecular weight of adiponectin from each species (an elephant seal was assumed to be exactly 26 kDa).

| Organism | Total Adiponectin (pmol/ml) | Fluid | Range Type | Assay Type | Patient Notes | Ref |
|---|---|---|---|---|---|---|
| human | 88.6 - 383.9 | plasma | 5th-95th percentile | ELISA | male, heart problems | [2] |
| | 159.4 - 842.7 | plasma | 5th-95th percentile | ELISA | male, healthy | [2] |
| | 30.0 - 100.0 | plasma | ± S.D. | MRM | male and female, young | [4] |
| | 110 | serum | | MRM | pool of 4 samples | [7] |
| | 11.9 - 37.3 | plasma | | MRM -2 peptide | mixed gender pool | [3] |
| | 0.8 - 5.0 | plasma | | MRM -2 peptide | mixed gender pool | [10] |
| | 371.0 - 1162.3 | unspecified | IQR | ELISA | mixed gender and race | [11] |
| | 251.8 - 611.0 | serum | ± S.E. | ELISA | mixed gender and race | [8] |
| | 71.9 - 643.6 | plasma | min and max | ELISA | healthy volunteers | [1] |
| | 329.4 - 427.8 | plasma | ± S.D. | ELISA | lean humans | [13] |
| dog | 227.6 - 2314.0 | plasma | min and max | ELISA | lean and obese dogs | [12] |
| rat | 60.6 - 272.6 | plasma | ± S.E. | ELISA | control and rats on diet | [15] |
| mouse | 231.3 - 1100.4 | serum | min and max | ELISA | SMXA RI strains | [6] |
| horse | 42.3 - 80.8 | plasma | ± S.E. | RIA | fit and unfit | [5] |
| cow | 765.3 - 1530.6 | serum | approx min and max | ELISA | pregnant or lactating dairy cows | [9] |
| cat | 174.7 - 266.1 | plasma | ± S.E. | ELISA | high and low carb diets | [12] |
| elephant seal | 10.8 - 16.2 | plasma | ± S.D. | ELISA | | [14] |


[1] Y. Arita, S. Kihara, N. Ouchi, M. Takahashi, K. Maeda, J. Miyagawa, *et al.*, Paradoxical decrease of an adipose-specific protein, adiponectin, in obesity. Biochem Biophys Res Commun. 257 (1999) 79-83.

[2] C. Caselli, O. Melaiu, M. Maltinti, S. Del Ry, M. Cabiati, T. Prescimone, *et al.*, A methodological reappraisal of total and high molecular weight adiponectin determination in human peripheral circulation: comparison of four immunometric assays. Clin Chem Lab Med. 48 (2010) 561-568.

[3] D. Domanski, A.J. Percy, J. Yang, A.G. Chambers, J.S. Hill, G.V. Freue, *et al.*, MRM-based multiplexed quantitation of 67 putative cardiovascular disease biomarkers in human plasma. Proteomics. 12 (2012) 1222-1243.

[4] B. Garcia-Bailo, D.R. Brenner, D. Nielsen, H.J. Lee, D. Domanski, M. Kuzyk, *et al.*, Dietary patterns and ethnicity are associated with distinct plasma proteomic groups. Am J Clin Nutr. 95 (2012) 352-361.

[5] M.E. Gordon, K.H. McKeever, C.L. Betros, H.C. Manso Filho, Plasma leptin, ghrelin and adiponectin concentrations in young fit racehorses versus mature unfit standardbreds. Vet J. 173 (2007) 91-100.

[6] M. Kobayashi, T. Ohno, T. Kawada, H. Ikegami, M. Nishimura, F. Horio, Serum adiponectin concentration: its correlation with diabetes-related traits and quantitative trait loci analysis in mouse SMXA recombinant inbred strains. Biosci Biotechnol Biochem. 70 (2006) 677-683.

[7] S. Lin, T.A. Shaler, C.H. Becker, Quantification of intermediate-abundance proteins in serum by multiple reaction monitoring mass spectrometry in a single-quadrupole ion trap. Anal Chem. 78 (2006) 5762-5767.

[8] A. Mente, F. Razak, S. Blankenberg, V. Vuksan, A.D. Davis, R. Miller, *et al.*, Ethnic variation in adiponectin and leptin levels and their association with adiposity and insulin resistance. Diabetes Care. 33 (2010) 1629-1634.

[9] M. Mielenz, B. Mielenz, S.P. Singh, C. Kopp, J. Heinz, S. Haussler, *et al.*, Development, validation, and pilot application of a semiquantitative Western blot analysis and an ELISA for bovine adiponectin. Domest Anim Endocrinol (2012).

[10] A.J. Percy, A.G. Chambers, J. Yang, D.B. Hardie, C.H. Borches. Advances in multiplexed MRM-based protein biomarker quantitation toward clinical utility. Biochim Biophys Acta (2013) [epub ahead of print].

[11] L.J. Rasmussen-Torvik, C.L. Wassel, J. Ding, J. Carr, M. Cushman, N. Jenny, *et al.*, Associations of body mass index and insulin resistance with leptin, adiponectin, and the leptin-to-adiponectin ratio across ethnic groups: the Multi-Ethnic Study of Atherosclerosis (MESA). Ann Epidemiol. 22 (2012) 705-709.

[12] H.Y. Tan, J.S. Rand, J.M. Morton, L.M. Fleeman, P.J. Armstrong, M. Coradini, *et al.*, Adiponectin profiles are affected by chronic and acute changes in carbohydrate intake in healthy cats. Gen Comp Endocrinol. 172 (2011) 468-474.

[13] K.R. Verkest, J.S. Rand, L.M. Fleeman, J.M. Morton, A.A. Richards, F.J. Rose, *et al.*, Distinct adiponectin profiles might contribute to differences in susceptibility to type 2 diabetes in dogs and humans. Domest Anim Endocrinol. 41 (2011) 67-73.

[14] J.A. Viscarra, C.D. Champagne, D.E. Crocker, R.M. Ortiz, 5'AMP-activated protein kinase activity is increased in adipose tissue of northern elephant seal pups during prolonged fasting-induced insulin resistance. J Endocrinol. 209 (2011) 317-325.

[15] M. Zhu, J. Miura, L.X. Lu, M. Bernier, R. DeCabo, M.A. Lane, *et al.*, Circulating adiponectin levels increase in rats on caloric restriction: the potential for insulin sensitization. Exp Gerontol. 39 (2004) 1049-1059.
