# Supplementary figures and images for: Ratiometric Measurements of Adiponectin by Mass Spectrometry in Bottlenose Dolphins (Tursiops truncatus) with Iron Overload Reveal an Association with Insulin Resistance and Glucagon
Source: Front Endocrinol (Lausanne). 2013 Sep 20;4:132. doi: 10.3389/fendo.2013.00132 (PMC3778387; doi:10.3389/fendo.2013.00132)

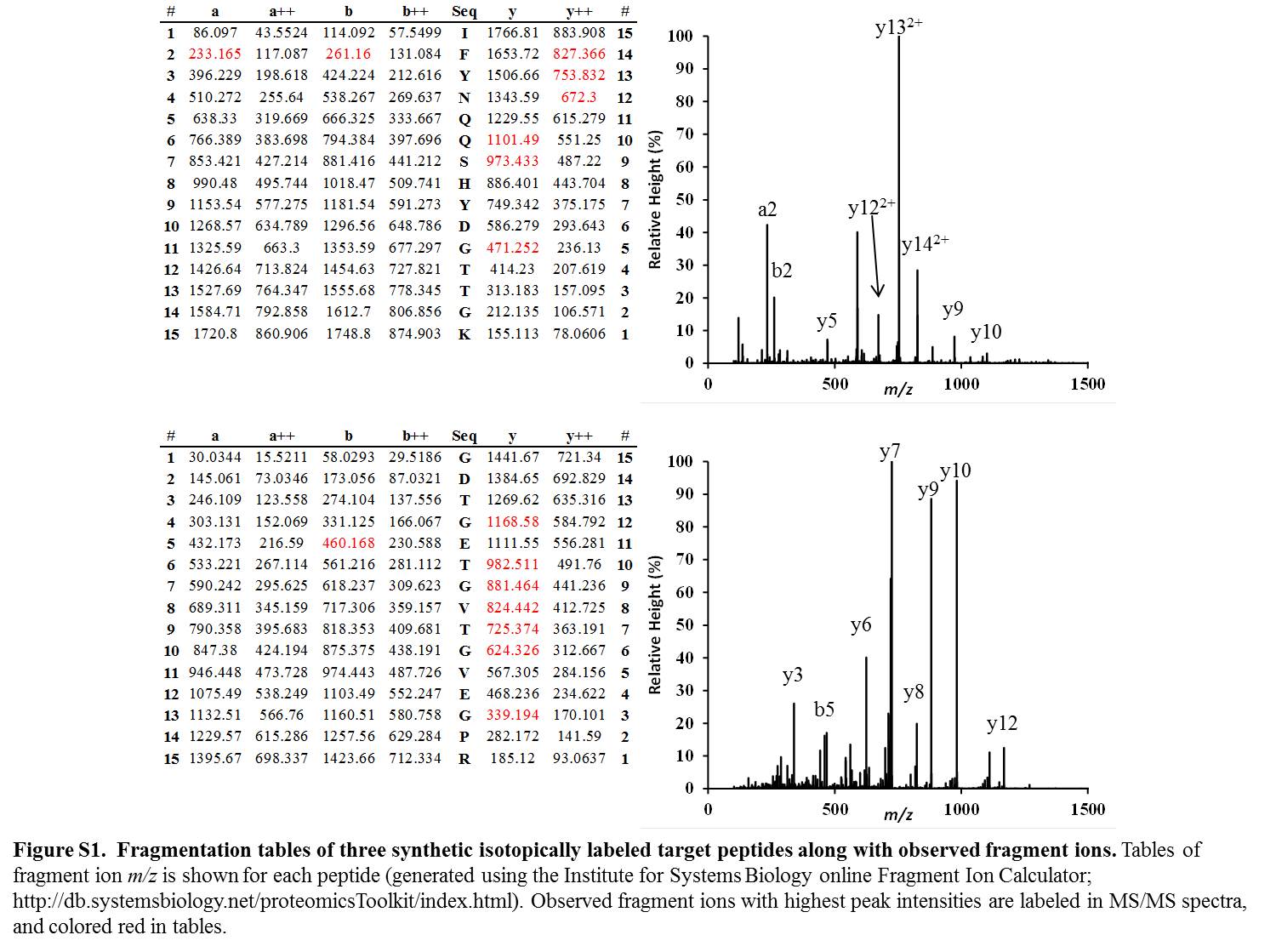

Supplement: Figure S1 — Fragmentation tables of three synthetic isotopically labeled target peptides along with observed fragment ions. Tables of fragment ion m/z is shown for each peptide (generated using the Institute for Systems Biology online Fragment Ion Calculator; http://db.systemsbiology.net/proteomicsToolkit/index.html). Observed fragment ions with highest peak intensities are labeled in MS/MS spectra, and colored red in tables. [file 65176_Janech_DataSheet1.ZIP › 65176_Janech_Figure_S1.JPEG]

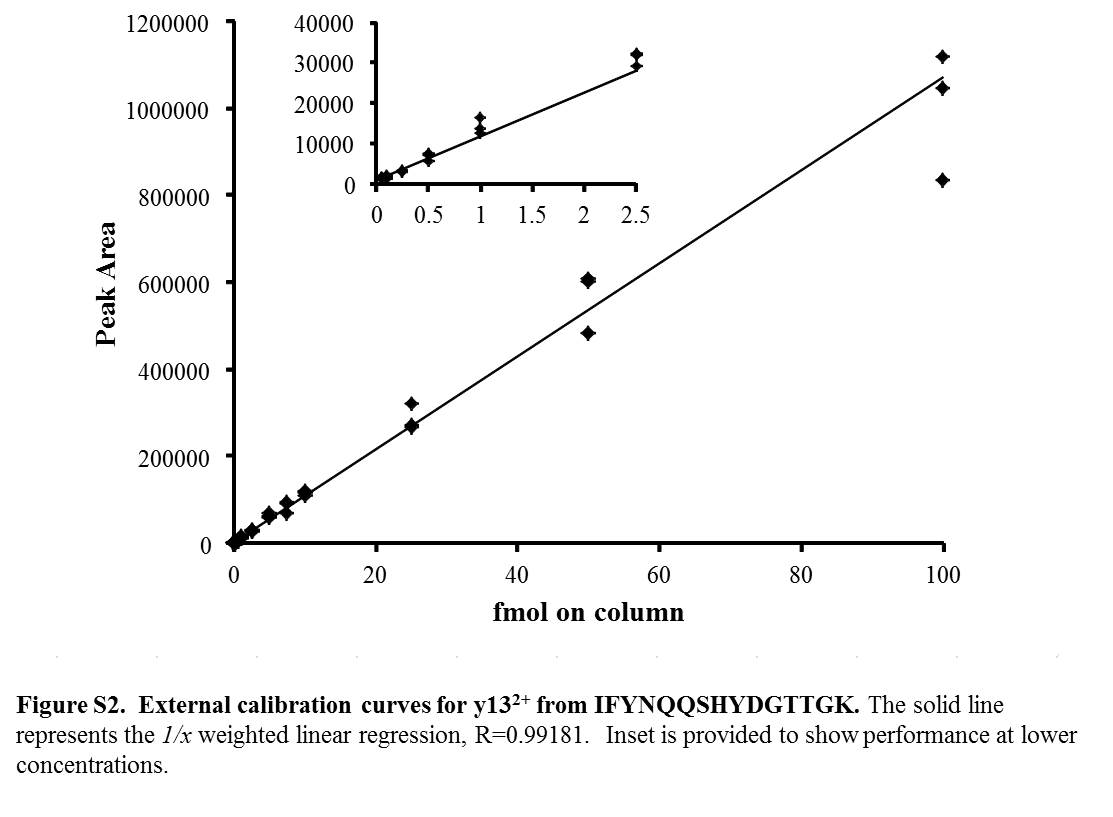

Supplement: Figure S1 — Fragmentation tables of three synthetic isotopically labeled target peptides along with observed fragment ions. Tables of fragment ion m/z is shown for each peptide (generated using the Institute for Systems Biology online Fragment Ion Calculator; http://db.systemsbiology.net/proteomicsToolkit/index.html). Observed fragment ions with highest peak intensities are labeled in MS/MS spectra, and colored red in tables. [file 65176_Janech_DataSheet1.ZIP › 65176_Janech_Figure_S2.JPEG]

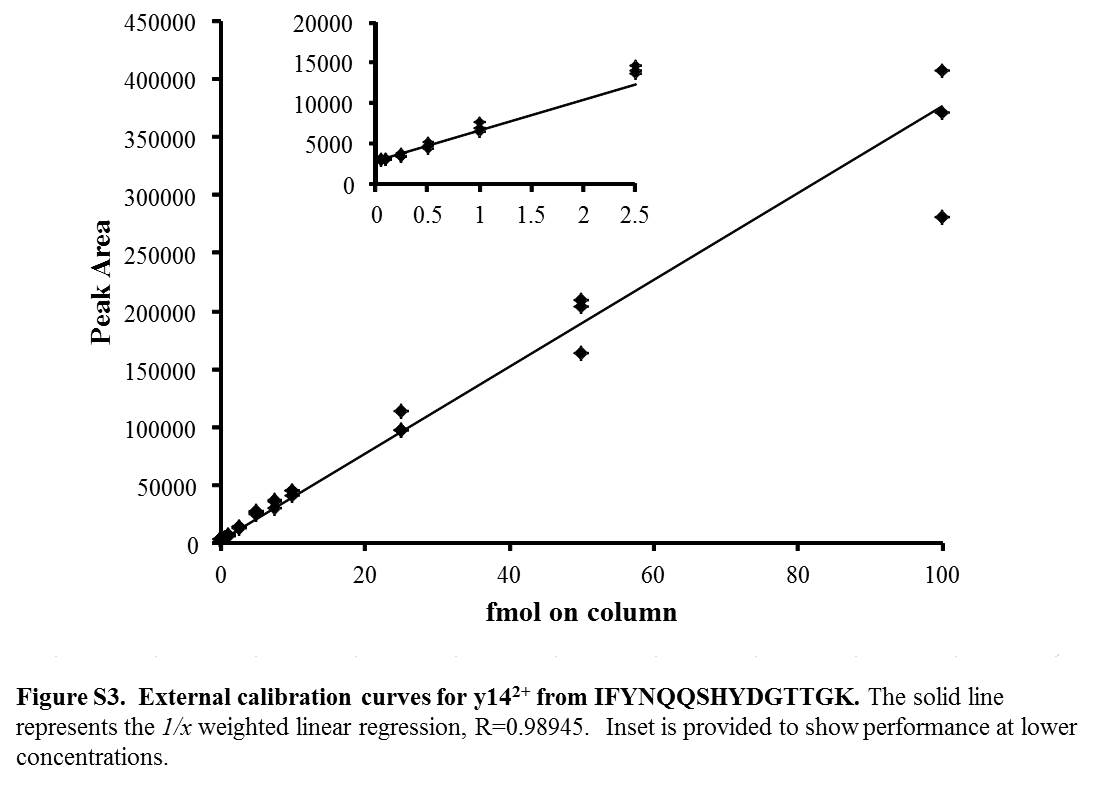

Supplement: Figure S1 — Fragmentation tables of three synthetic isotopically labeled target peptides along with observed fragment ions. Tables of fragment ion m/z is shown for each peptide (generated using the Institute for Systems Biology online Fragment Ion Calculator; http://db.systemsbiology.net/proteomicsToolkit/index.html). Observed fragment ions with highest peak intensities are labeled in MS/MS spectra, and colored red in tables. [file 65176_Janech_DataSheet1.ZIP › 65176_Janech_Figure_S3.JPEG]

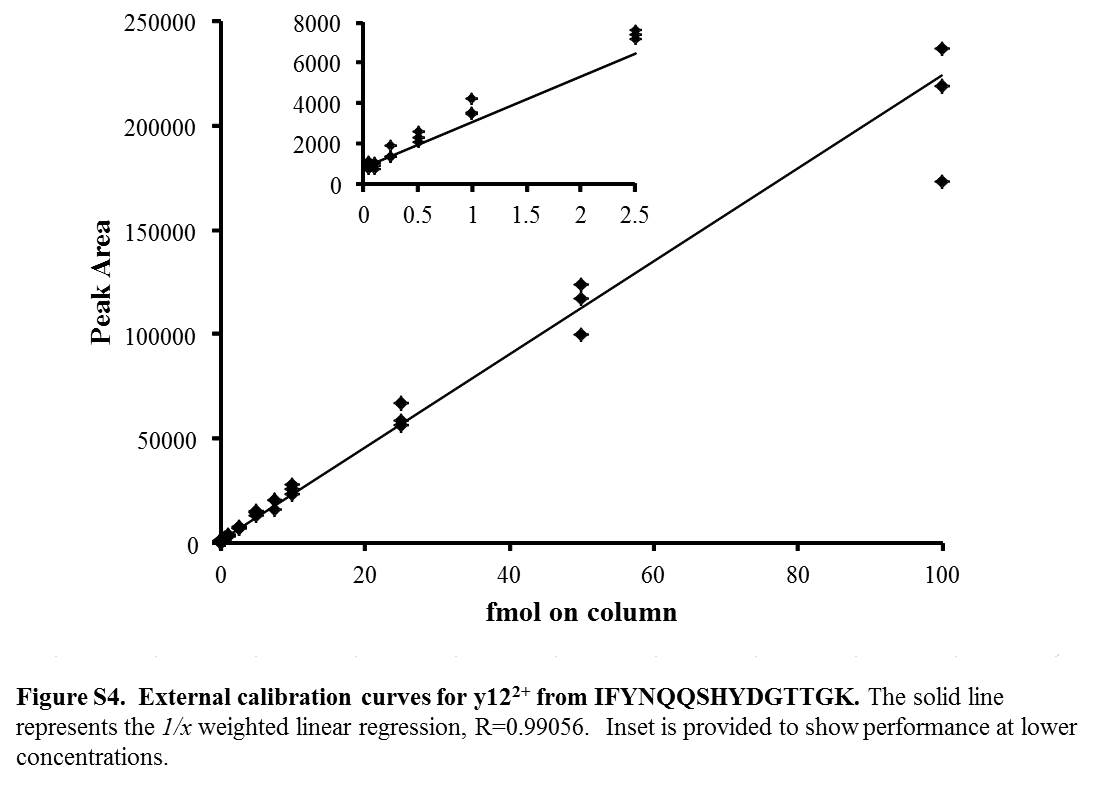

Supplement: Figure S1 — Fragmentation tables of three synthetic isotopically labeled target peptides along with observed fragment ions. Tables of fragment ion m/z is shown for each peptide (generated using the Institute for Systems Biology online Fragment Ion Calculator; http://db.systemsbiology.net/proteomicsToolkit/index.html). Observed fragment ions with highest peak intensities are labeled in MS/MS spectra, and colored red in tables. [file 65176_Janech_DataSheet1.ZIP › 65176_Janech_Figure_S4.JPEG]

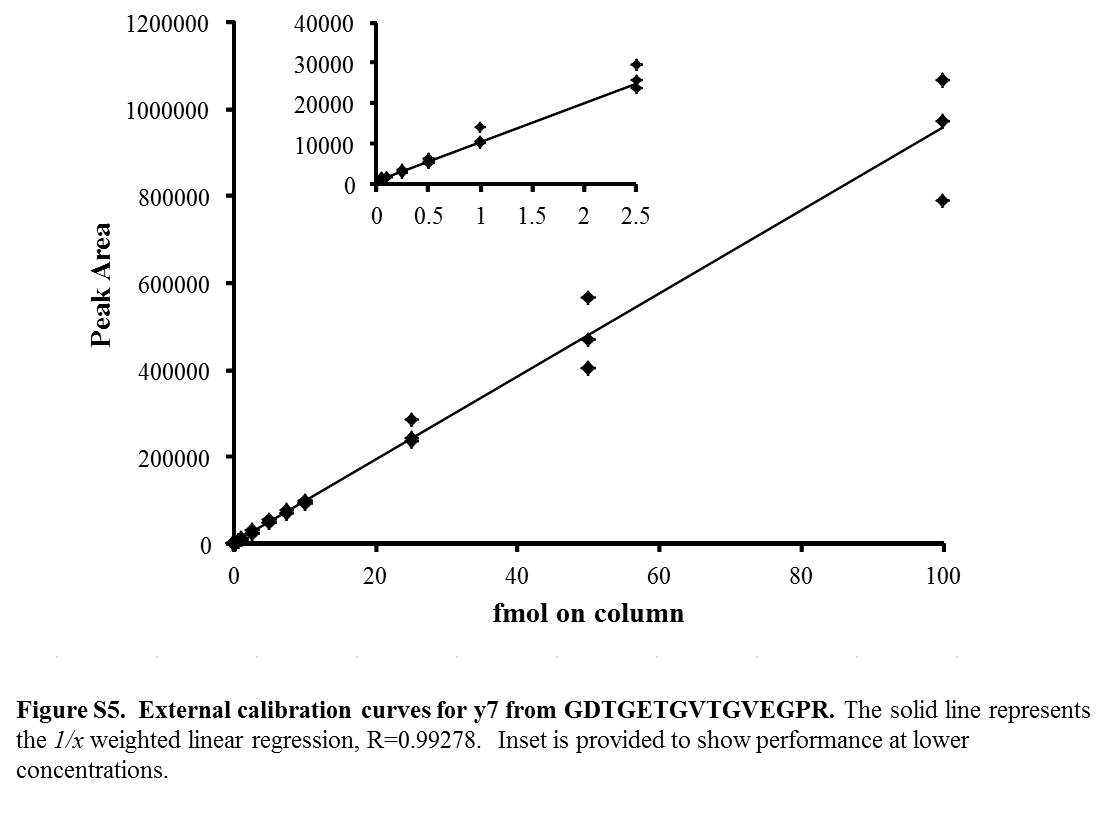

Supplement: Figure S1 — Fragmentation tables of three synthetic isotopically labeled target peptides along with observed fragment ions. Tables of fragment ion m/z is shown for each peptide (generated using the Institute for Systems Biology online Fragment Ion Calculator; http://db.systemsbiology.net/proteomicsToolkit/index.html). Observed fragment ions with highest peak intensities are labeled in MS/MS spectra, and colored red in tables. [file 65176_Janech_DataSheet1.ZIP › 65176_Janech_Figure_S5.JPEG]

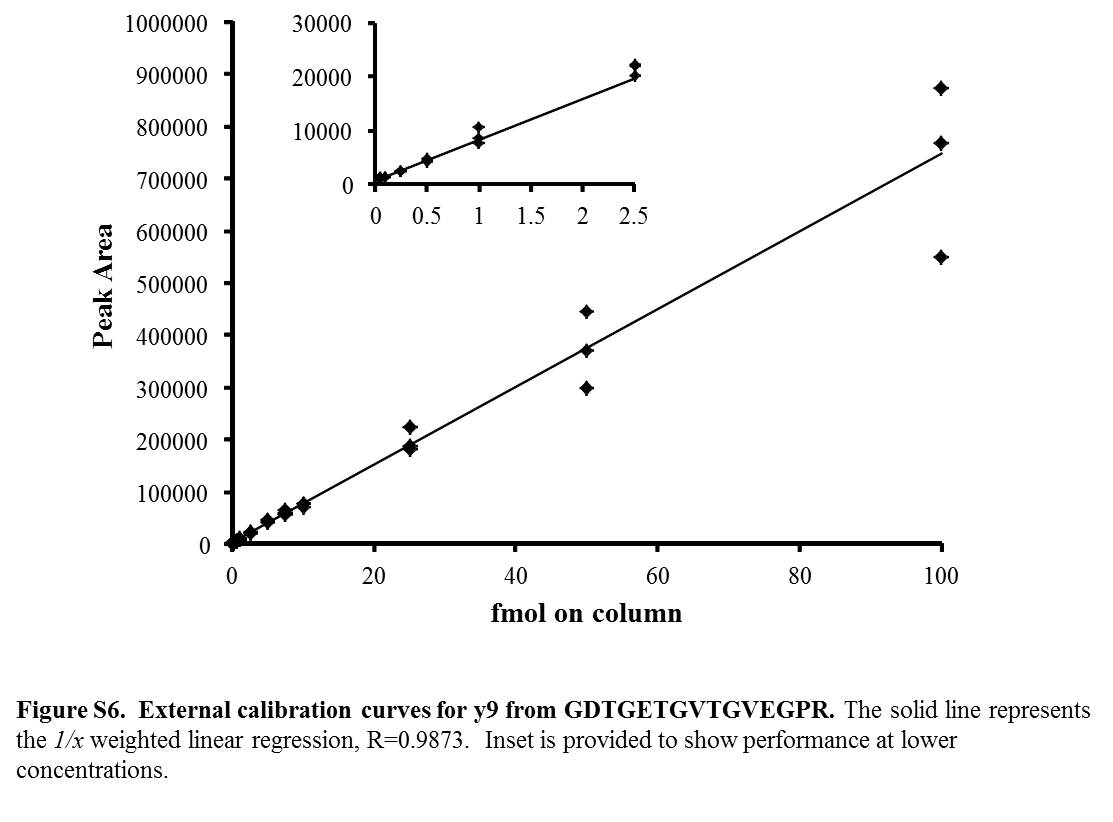

Supplement: Figure S1 — Fragmentation tables of three synthetic isotopically labeled target peptides along with observed fragment ions. Tables of fragment ion m/z is shown for each peptide (generated using the Institute for Systems Biology online Fragment Ion Calculator; http://db.systemsbiology.net/proteomicsToolkit/index.html). Observed fragment ions with highest peak intensities are labeled in MS/MS spectra, and colored red in tables. [file 65176_Janech_DataSheet1.ZIP › 65176_Janech_Figure_S6.JPEG]

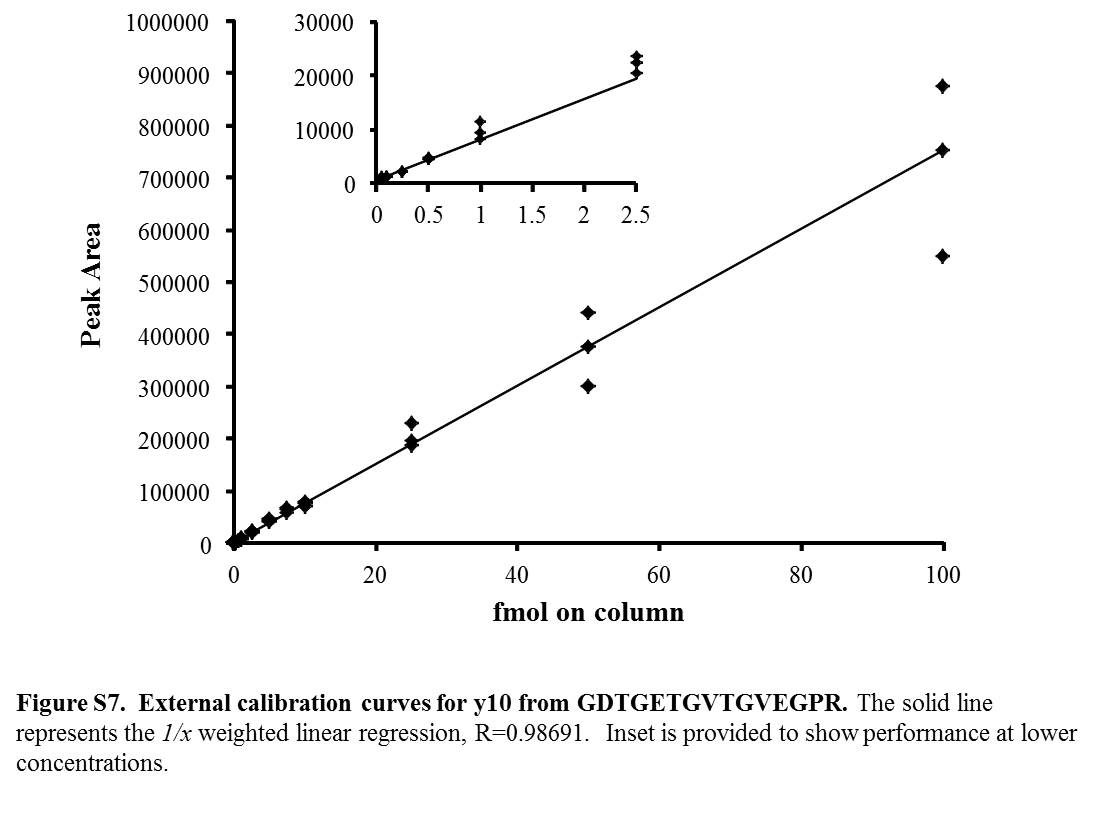

Supplement: Figure S1 — Fragmentation tables of three synthetic isotopically labeled target peptides along with observed fragment ions. Tables of fragment ion m/z is shown for each peptide (generated using the Institute for Systems Biology online Fragment Ion Calculator; http://db.systemsbiology.net/proteomicsToolkit/index.html). Observed fragment ions with highest peak intensities are labeled in MS/MS spectra, and colored red in tables. [file 65176_Janech_DataSheet1.ZIP › 65176_Janech_Figure_S7.JPEG]
